# Comparison of Procedure and Fluoroscopy Time Between Left Bundle Branch Area Pacing and Right Ventricular Pacing for Bradycardia: The Learning Curve for the Novel Pacing Strategy

**DOI:** 10.3389/fcvm.2021.695531

**Published:** 2021-09-23

**Authors:** Zhao Wang, Haojie Zhu, Xiaofei Li, Yan Yao, Zhimin Liu, Xiaohan Fan

**Affiliations:** Department of Cardiology, Fuwai Hospital, National Center for Cardiovascular Diseases, Chinese Academy of Medical Sciences, Peking Union Medical College, Beijing, China

**Keywords:** left bundle branch area pacing, right ventricular pacing, learning curve, procedure duration, fluoroscopy time

## Abstract

**Background:** Left bundle branch area pacing (LBBAP) is a novel physiological pacing approach.

**Objective:** To assess learning curve for LBBAP and compare the procedure and fluoroscopy time between LBBAP and right ventricular pacing (RVP).

**Methods:** Consecutive bradycardia patients who underwent LBBAP or RVP were prospectively recruited from June 2018 to June 2020. The procedure and fluoroscopy time for ventricular lead placement, pacing parameters, and periprocedural complications were recorded. Restricted cubic splines were used to fit learning curves for LBBAP.

**Results:** Left bundle branch area pacing was successful in 376 of 406 (92.6%) patients while 313 patients received RVP. Learning curve for LBBAP illustrated initial (1–50 cases), improved (51–150 cases), and stable stages (151–406 cases) with gradually increased success rates (88.0 vs. 90.0 vs. 94.5%, *P* = 0.106), steeply decreased median procedure (26.5 vs. 14.0 vs. 9.0min, *P* < 0.001) and fluoroscopy time (16.0 vs. 6.0 vs. 4.0min, *P* < 0.001), and shortened stimulus to left ventricular activation time (Sti-LVAT; 78.7 vs. 78.1 vs. 71.2 ms, *P* < 0.001). LBBAP at the stable stage showed longer but close median procedure (9.0 vs. 6.9min, *P* < 0.001) and fluoroscopy time (4.0 vs. 2.8min, *P* < 0.001) compared with RVP.

**Conclusion:** The procedure and fluoroscopy time of LBBAP could be reduced significantly with increasing procedure volume and close to that of RVP for an experienced operator.

## Introduction

Traditional right ventricular pacing (RVP) has been extensively used in clinical practice for more than 50 years. However, RV apex pacing (RVAP) can produce a deleterious effect on cardiac function and consequently increase the risk of heart failure and atrial fibrillation, especially in patients with a high burden of ventricular pacing (1). Pacing the right ventricular septum (RVSP) or outflow tract does not present superiority to RVAP (2, 3). Biventricular pacing can maintain interventricular electromechanical synchrony and has been proposed as an alternative to RVP in patients with heart failure and atrioventricular block (AVB) (4).

His bundle pacing (HBP) has been the most physiological pacing modality since 2000 (5). However, routine application of HBP has been limited in specific subgroups due to the high capture threshold, low sensing amplitude, potential risk of loss of capture, and a steep learning curve (6). Left bundle branch area pacing (LBBAP), first reported by Huang et al. (7) has emerged as a promising physiological pacing modality with stable low threshold and other pacing parameters. Recently, the middle- and long-term feasibility and safety of LBBAP have been demonstrated in patients with symptomatic bradycardia or advanced heart failure (8, 9). Compared with HBP, LBBAP could achieve a similar paced QRS duration (pQRSd), success rate, and better pacing parameters with significantly shorter procedure duration and fluoroscopy time (10). Compared with RVAP or RVSP, LBBAP presents a significantly narrower pQRSd, similar pacing parameters, and significantly longer procedure and fluoroscopy time (8, 11, 12). However, most studies reported their experience of the LBBAP procedure at the initial stage. Few studies focused on learning curves for LBBAP. Whether the procedure duration of LBBAP after a series of cases with currently available implantation tools could be comparable to RVP has not been investigated. Therefore, the present study aimed to (1) fit learning curves for LBBAP indicated by procedure and fluoroscopy time; (2) compare the procedure and fluoroscopy time, electrophysiological parameters, and periprocedural complications between LBBAP at different learning stages and RVP.

## Methods

### Study Populations

We prospectively enrolled consecutive patients who attempted LBBAP or RVP procedures in our working group at Fuwai Hospital from June 2018 to June 2020. All patients had symptomatic bradycardia and were indicated for pacemaker implantation according to the current American College of Cardiology/American Heart Association/Heart Rhythm Society guidelines (13). Patients were excluded if they were younger than 18 years or indicated for cardiac resynchronization therapy (CRT) or implantable cardioverter-defibrillator, or underwent pacemaker replacement or upgrade with existing lead, or the procedures were not performed by our group. All participants provided written informed consent, and the institutional review board of Fuwai hospital approved this study.

### Procedures

All procedures were performed under local anesthesia. Preventive antibiotics were administered intravenously half an hour before the procedure. Venous access was usually obtained via the left axillary vein, sometimes via the right axillary vein due to various reasons.

**LBBAP:** All LBBAP procedures were performed by an experienced operator in RVP and HBP. Our previous study has described the LBBAP procedure by using the single-lead technique in the initial stage (14). Briefly, we first mapped the His bundle electrogram from the lead tip and then moved the tip 1.5–2 cm toward the RV apex with the tricuspid annulus as a landmark. And then the ideal screwing site was identified by pace mapping. After nearly 20 procedures, His mapping was discarded, and the 3830 lead was directly advanced to the RV septal area 1.5–2 cm from tricuspid annulus, and then pace mapping was used to find the target-screwing site. The lead tip was quickly screwed into the septum with approximately 5–6 clockwise rotations. As the lead was screwed deeper, detailed pacing tests were performed frequently. The surface 12-lead electrocardiogram (ECG), the intracardiac electrogram (IEGM), and fluoroscopy imaging were simultaneously monitored during the procedure, and left bundle branch (LBB) potential was recorded. Pacing stimulus to left ventricular activation time (Sti-LVAT) in lead V5 was measured at low (at 2V/0.4 ms) and high (at 5V/0.4 ms) outputs. Our method without His mapping was similar to the simplified nine-partition method (15, 16) and was performed in all the rest patients. The criteria of successful LBBAP were defined per previously published criteria (17). If successful LBBAP could not be achieved after five attempts or fluoroscopy duration exceeded 20 min, LVSP was then preferred to achieve a relatively narrow QRSd, with the lead positioned in the mid-LV septum. An electrophysiology recording system (Bard/Boston Scientific, Lowell, MA, USA) was used to monitor and record the IEGM in 90.6% of patients, while a surface 12-lead ECG was used alone in 9.4% of all patients with LBBAP.

**RVP:** All RVP procedures were performed by two experienced operators. The active-fixation pacing lead was positioned at the RV septum. Fluoroscopic radiographs from 45° left anterior oblique (LAO) were applied to confirm the RV lead position.

### Data Collection and Device Programming

Baseline clinical data were collected, such as demographic characteristics, medical histories, pacing indications, ECG, and echocardiographic evaluation parameters. For LBBAP, the procedure and fluoroscopy time counting began when the C315 HIS sheath was advanced and ended when the LBBAP or LVSP was achieved. For RVP, the procedure and fluoroscopy time were defined as the duration from the beginning of delivery sheath to the end of successful placement of the ventricular lead. Pacing parameters (capture threshold, impedance, and sensing amplitude) were recorded. ECG parameters were measured at a sweep speed of 100 mm/s on electrophysiology recording systems, such as LBB potential to ventricle interval (P-V interval), Sti-LVAT, pQRSd, QRS axis deviation, and QRS transition zone. Periprocedural complications were documented, such as lead dislodgement and revision, lead perforation, pacing system infection, and other device-related complications.

Depending on the intrinsic atrioventricular (AV) conduction interval and conduction system disease, individualized AV delay was programmed. Automatic AV search algorithm was routinely turned on in patients with intact AV conduction to avoid unnecessary ventricular pacing.

### Statistical Analysis

Continuous variables are presented as mean ± SD or median with interquartile range according to the normal distribution of data. The means or medians are compared using the Student's *t*-test or analysis of variance or the Kruskal–Wallis H test. Categorical variables are expressed as frequency or percentage and compared using chi-square or Fisher exact test. We used restricted cubic splines (RCS) with four knots at the 5th, 35th, 65th, and 95th centiles to flexibly model and visualize the correlations between procedure and fluoroscopy time and numbers of procedures. Based on the learning curve, all LBBAP procedures were divided into three groups: initial (1–50 cases), improved (51–150 cases), and stable stages (151–406 cases). A two-tailed *P* < 0.05 was considered statistically significant. Statistical analysis was performed using R version 3.5.1 (R Foundation for Statistical Computing, Vienna, Austria).

## Results

### Baseline Clinical Characteristics

A total of 406 patients who received LBBAP procedures during the study period were included. The mean age was 64.9 ± 14.3 years old, and male patients accounted for 48.5%. Indications for pacemaker implantation included sinus node dysfunction (SND) in 39.7% of patients and AVB in 60.3% of patients. Baseline Baseline left or right bundle branch block (LBBB or RBBB) was present in 10.5 and 23.4% of patients. Other baseline clinical features are summarized in Table 1.

**Table 1:** Baseline clinical and demographic features of patients attempting LBBAP.

**Variables**	**LBBAP** **(***n*** = 406)**
Age	64.9 ± 14.3
Male	197(48.5%)
Hypertension	244(60.1%)
Diabetes	79 (19.5%)
Atrial fibrillation	178(43.8%)
Paroxysmal	104(58.4%)
Persistent	74 (41.6%)
CAD	76 (18.7%)
Valvular heart disease	35 (8.6%)
Hypertrophic cardiomyopathy	10 (2.5%)
Baseline electrocardiogram	
Heart rate	54.7 ± 17.5
QRS duration	112 ± 24.1
Left bundle branch block	43 (10.5%)
Right bundle branch block	95 (23.4%)
Pacing indications	
AVB	245(60.3%)
SND	161(39.7%)
Baseline Echocardiography	
LAD	40.2 ± 8.45
LVEDD	48.6 ± 6.91
LVEF	61.2 ± 7.27
IVS	9.82 ± 1.93
Type of device	
Double-chamber PM	341(84.0%)
Single-chamber PM	65 (16.0%)

*Data are presented as mean ± SD for continuous variables and number and percentages for categorical variables*.

*LBBAP, left bundle branch area pacing; CAD, coronary artery disease; AVB, atrioventricular block; SND, sinus node dysfunction; LAD, left atrium diameter; LVEDD, Left ventricular end-diastolic diameter; LVEF, Left ventricular ejection fraction; IVS, interventricular septum; PM, pacemaker*.

### Implantation Outcomes and Learning Curves of LBBAP

**Figure 1:**
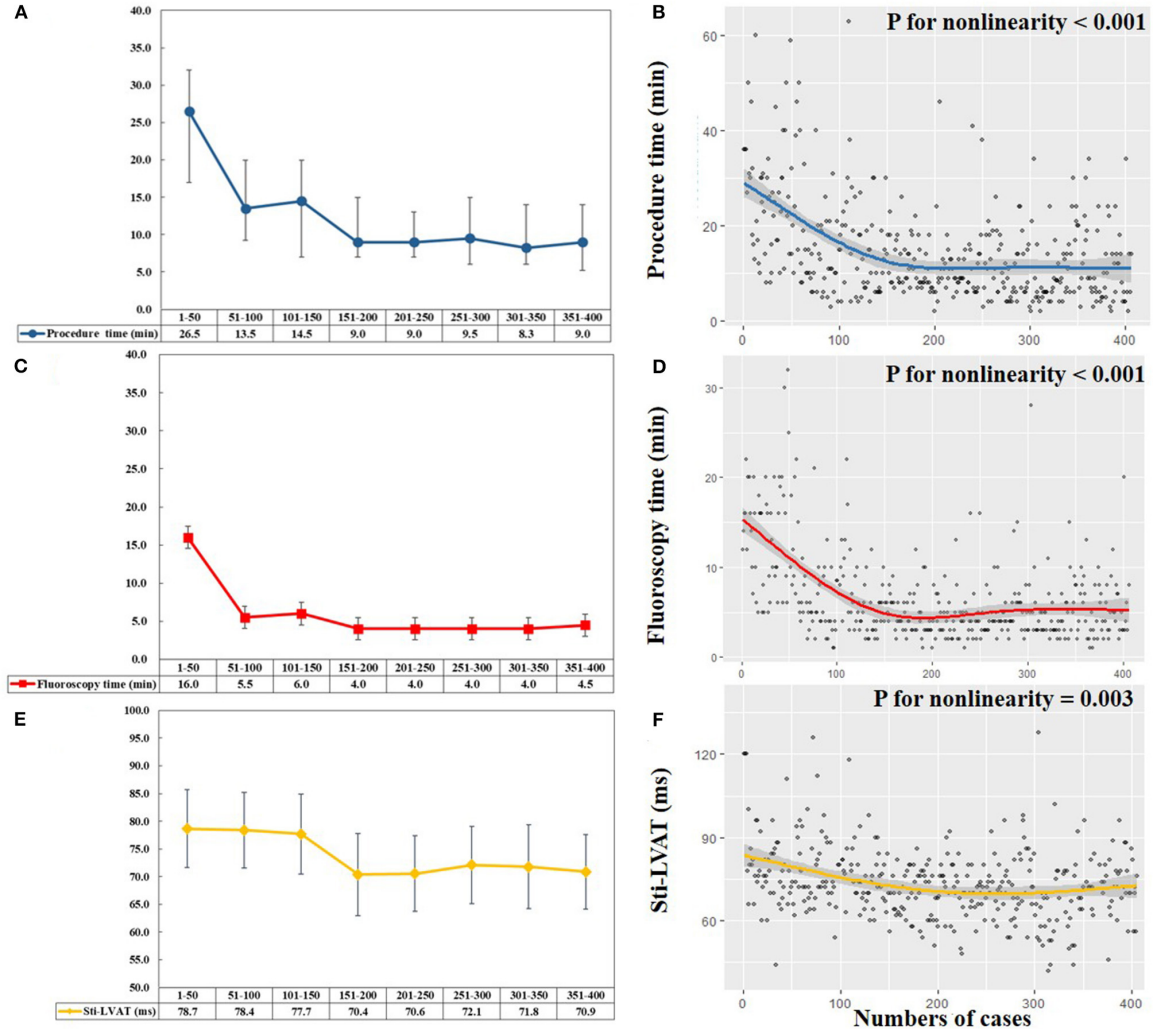
Learning curves of left bundle branch area pacing (LBBAP). **(A,C)** Median procedure time (blue line) and fluoroscopy time (red line) of per 50 consecutive patients attempting LBBAP. (**B,D)** Scatterplots and predicted learning curves of LBBAP indicated by procedure time (blue line) and fluoroscopy time (red line). The curves rapidly decreased until the 150th procedure and then reached a plateau, with the procedure time of 9min and fluoroscopy time of 5min (both *P* for nonlinearity < 0.001). (**E)** Mean Sti-LVAT (yellow line) at the high output (5 V/0.4 ms) of per 50 consecutive patients attempting LBBAP. (**F)** Scatterplot and predicted learning curve of LBBAP indicated by Sti-LVAT (yellow line). The curve plateaued after approximately 200 procedures (*P* for nonlinearity = 0.003). Sti-LVAT, Stimulus to left ventricular activation time.

Figure 1 shows trends in procedural performance reflected by procedure duration, fluoroscopy time, and Sti-LVAT at high output. We first divided all patients into eight groups (every 50 cases in one group) across the study period. The median procedure duration and fluoroscopy time of each group were listed (Figures 1A,C). In the first 50 cases, the median procedure time and fluoroscopy time for ventricular lead implantation were 26.5 and 16.0 min, respectively. The median time was markedly decreased in the following 100 procedures (from 51 to 150). Since the 151st procedure, the median procedure and fluoroscopy time reached a relatively low plateau and ranged from 8.3 to 9.5 min and 4.0 to 4.5 min. Figures 1B,D visualizes the association between the procedure or fluoroscopy time and numbers of LBBAP procedures. Both the predicted procedure duration and fluoroscopy time dropped off sharply until around the 150th case and became relatively stable afterward (Both *P* for nonlinearity < 0.001). The steepest part of learning curves appeared to be over the first 50 cases. The procedure and fluoroscopy time were improved over the following 100 cases (from 51 to 150) and stabilized after 150 cases (from 151 to 406).

The changing trends of Sti-LVAT at the high output are shown in Figures 1E,F. During the first 150 procedures, the mean Sti-LVAT was stable at 77.7–78.7 ms. After 150 procedures, the mean Sti-LVAT was markedly shortened and plateaued at 70.4–72.1 ms. Figure 1F shows the predicted Sti-LVAT curve (*P* for nonlinearity = 0.003).

### ECG and Pacing Parameters at Different LBBAP Stages

Based on learning curves of LBBAP, three step-by-step stages were identified: initial stage (*n* = 50, procedure 1–50), improved stage (*n* = 100, procedure 51–150), and stable stage (*n* = 256, procedure 151–406). As shown in Table 2, the success rate of LBBAP was 92.6% in overall patients, and gradually increased along three stages (88.0 vs. 90.0 vs. 94.5%, *P* = 0.106). The mean Sti-LVAT at the stable stage was the shortest at the high output (71.2 ± 11.6 ms) and low output (74.3 ± 16.2 ms). The mean LBB capture threshold and the mean pQRSd did not differ among the three stages (*P* > 0.05). The mean numbers of attempts were significantly different among the three stages (2.1 ± 0.7 vs. 1.7 ± 0.8 vs. 1.2 ± 0.5, *P* < 0.001). With accumulated experience, we attempted once to achieve successful LBBAP in 80% of cases in the stable stage, twice in 10% of cases, and three times or more in the rest of cases. The median procedure (26.5 vs. 14.0 vs. 9.0 min, *P* < 0.001) and fluoroscopy time (16.0 vs. 6.0 vs. 4.0 min, *P* < 0.001) rapidly decreased from initial to stable stages.

**Table 2:** Pacing and procedural parameters in patients attempting LBBAP.

**Variables**	**Overall** **(***n*** = 406)**	**Initial stage** **(***n*** = 50)**	**Improved stage** **(***n*** = 100)**	**Stable stage** **(***n*** = 256)**	***P*** **-value**
IEGM, n (%)	368 (90.6%)	46 (92%)	94 (94%)	228 (89.1%)	0.205
Successful LBBAP, n (%)	376 (92.6%)	44 (88.0%)	90 (90.0%)	242 (94.5%)	0.106
LBB potential, n (%)^*^	256 (68.1%)	31 (70.5%)	74 (82.2%)	151 (62.4%)	0.040
P-V interval, ms	27.7 ± 4.7	29.6 ± 6.7	27.1 ± 4.9	27.7 ± 4.2	0.78
Sti-LVAT at 5V/0.4 ms, ms	73.9 ± 13.4	78.7 ± 16.4	78.1 ± 14.2	71.2 ± 11.6	<0.001
Sti-LVAT at 2V/0.4 ms, ms	76.7 ± 15.4	83.4 ± 13.5	79.2 ± 14.2	74.3 ± 16.2	<0.001
Anodal capture at 2V/0.4 ms, n (%)^*^	366 (97.3%)	42 (95.5%)	87 (96.7%)	237 (97.9%)	0.879
Ring capture threshold, V/0.4 ms	1.04 ± 0.65	0.96 ± 0.53	1.06 ± 0.75	1.04 ± 0.64	0.696
Paced QRSd, ms	114 ± 10.7	117 ± 11.5	114 ± 9.5	114 ± 10.4	0.303
LBB capture threshold, V/0.4 ms	0.64 ± 0.21	0.65 ± 0.17	0.65 ± 0.21	0.64 ± 0.23	0.874
Impedance, Ω	783 ± 154	762 ± 144	773 ± 166	791 ± 157	0.420
R wave amplitude, mV	11.7 ± 6.1	11.5 ± 4.9	12.3 ± 9.3	11.4 ± 4.9	0.476
QRS axis^*^					0.548
Normal axis, n(%)	265 (70.5%)	23 (52.3%)	70 (77.8%)	172 (71.1%)	
Left axis deviation, n(%)	85 (22.6%)	11(25.0%)	16 (17.8%)	58 (24.0%)	
Right axis deviation, n(%)	26 (6.9%)	10 (22.7%)	4 (4.4%)	12 (5.0%)	
QRS transition zone^#^	3 (2, 3)	2 (2, 3)	3 (2, 3)	3 (2, 4)	0.248
Numbers of attempts	1.4 ± 0.6	2.1 ± 0.7	1.7 ± 0.8	1.2 ± 0.5	<0.001
Procedure time, min	11.0 (7.0, 18.8)	26.5 (17.0, 32.0)	14.0 (8.0, 20.0)	9.0 (6.0, 14.0)	<0.001
Fluoroscopy time, min	5.0 (3.0, 8.0)	16.0 (9.0, 18.0)	6.0 (4.0, 9.0)	4.0 (3.0, 6.0)	<0.001

*Data are presented as mean ± standard deviation or median (Q1, Q3) for continuous variables and frequency and percentages for categorical variables*.

*IEGM, intracardiac electrogram; LBBAP, left bundle branch area pacing; LBB, left bundle branch; P-V interval, interval from LBB potential to ventricle; Sti-LVAT, pacing stimulus to left ventricular activation time; QRSd, QRS duration*.

* *divided by successful LBBAP cases*.

# *each number represents the corresponding precordial lead*.

### Comparison Between LBBAP and RVP

A total of 313 patients received RVP during the same period. LBBAP was more frequently performed in patients with AVB (60.3 vs. 27.5%, *P* < 0.001; Table 3). Periprocedural complications did not differ between LBBAP and RVP (*P* = 0.658). One ventricular septal perforation and one lead dislodgement occurred soon after the LBBAP procedure at the initial stage. Both patients patients had no symptoms except for having features of pacing failure. Two lead dislodgements happened in the RVP group. All lead revisions were successful without further clinical symptoms or signs.

**Table 3:** Comparison of clinical characteristics and periprocedural complications between LBBAP and RVP.

**Variables**	**LBBAP** **(***n*** = 406)**	**RVP** **(***n*** = 313)**	***P*** **-value**
Age	64.9 ± 14.3	67.5 ± 12.2	0.573
Male	197 (48.5%)	150 (47.9%)	0.940
Pacing indications			<0.001
AVB	245 (60.3%)	86 (27.5%)	
SND	161 (39.7%)	227 (72.5%)	
Type of device			<0.001
Double-chamber PM	341 (84.0%)	297 (94.9%)	
Single-chamber PM	65 (16.0%)	16 (5.1%)	
Periprocedural complications			
Lead revision	2	2	0.658
Lead dislodgement	1	2	
Lead perforation	1	0	
Pericardial effusion	0	0	1
Pacing system infection	0	0	1
Pocket hematoma	0	0	1
Pneumothorax/hemothorax	0	0	1

*Data are presented as mean ± standard deviation for continuous variables and frequency and percentages for categorical variables*.

*LBBAP, left bundle branch area pacing; RVP, right ventricular pacing; AVB, atrioventricular block; SND, sinus node dysfunction; PM, pacemaker*.

**Figure 2:**
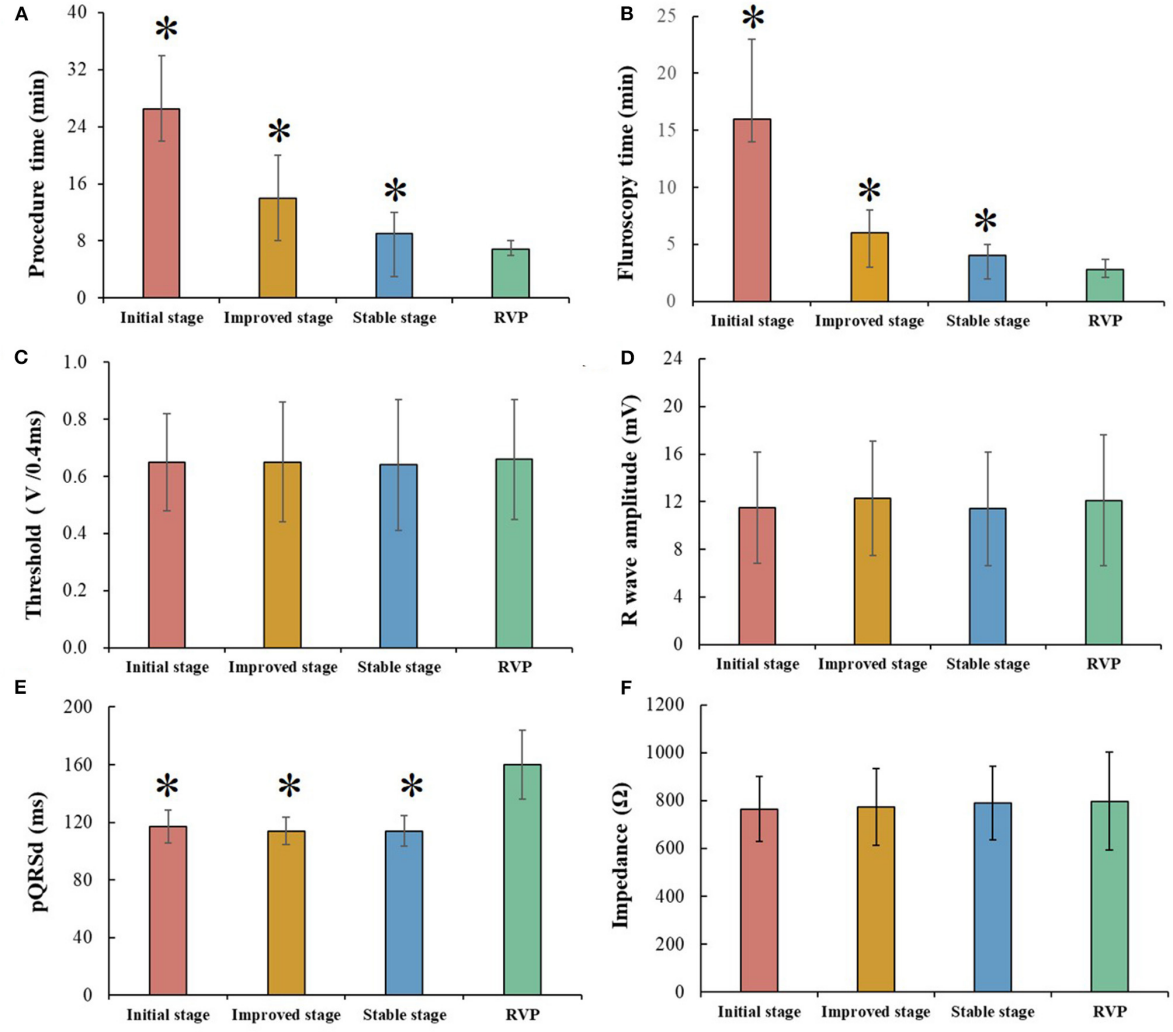
Comparison between different left bundle branch area pacing (LBBAP) stages and right ventricular pacing (RVP). (**A,B)** Procedure time and fluoroscopy time gradually decreased among three LBBAP stages but still longer than that of RVP. (**C,D,F)** Pacing parameters, including, capture threshold, R wave amplitude, and impedance, were comparable among three LBBAP stages and RVP. (**E)** LBBAP produced a narrower pQRSd than RVP; *P < 0.05 compared with RVP.

Figure 2 shows comparisons of procedural and pacing parameters between LBBAP at three stages and RVP. As compared with RVP, LBBAP at stable stage presented statistically longer procedure duration (9.0 vs. 6.7 min, *P* < 0.001) and fluoroscopy time (4.0 vs. 2.8 min, *P* < 0.001). However, RVP produced significantly wider pQRSd compared with that in three stages of successful LBBAP (160 ± 24.1 vs. 117 ± 11.5 vs. 114 ± 9.5 vs. 114 ± 10.4 ms, *P* < 0.001). Similar pacing parameters were observed between LBBAP at three stages and RVP (Figures 2 C,D,F).

## Discussion

This single-center study firstly demonstrated learning curves of LBBAP indicated by procedure duration and fluoroscopy time and made a comparison between LBBAP at three stages and RVP. The main findings of our study are as follows: (1) for operators who are adept at pacemaker implantation, the steepest part of learning curves for LBBAP was over the first 50 cases. The procedure and fluoroscopy time could be improved further over the following 100 cases and plateaued after 150 cases; (2) using the currently available implantation tools, the success rate of LBBAP could be 94.5% or more at the stable stage, and the fluoroscopy time for the ventricular lead placement could be as short as 4.0 min; (3) although LBBAP presented statistically significantly longer procedure and fluoroscopy time than RVP did, the absolute values were very close to that of RVP. Considering the advantages of LBBAP compared with RVP, our results indicate that LBBAP might be considered as the first choice in patients with a high burden of ventricular pacing to avoid the potential risk of cardiac dysfunction induced by RVP.

### Procedure and Fluoroscopy Time of LBBAP and the Learning Curve

Left bundle branch area pacing has been recently used as an alternative physiological pacing modality to HBP in some centers. Previous studies have reported fluctuated procedure and fluoroscopy time at different performing phases (8–11, 14, 18–20). The different definitions of procedure and fluoroscopy time might lead to significantly varied records. The procedure and fluoroscopy time in our study were defined as the duration of ventricular lead placement because the implant of atrial lead may confound the comparison between LBBAP and RVP. Chen et al. firstly reported a mean fluoroscopy time of 4.82 ± 3.37 min for the LBBAP procedure (21) and then presented a mean procedure duration of 18.0 ± 8.8 min and a mean fluoroscopy time of 3.9 ± 2.7 min for LBBAP implantation (18). Su et al. recently reported the largest single-center cohort study of LBBAP with a mean fluoroscopy time of 5.1 ± 4.6 min for lead placement and a mean procedure time of 86.4 ± 43.5 min (9). The median fluoroscopy time at the stable stage (4 [3.0, 6.0] min) in our study was consistent with previous results (3.9-5.1 min) (9, 18).

Different techniques applied to achieve LBBAP could also affect the procedure time. Huang et al. reported the “dual lead technique” with a mean fluoroscopy time ranging from 5.1 ± 4.6 min to 6.9 ± 2.5 min (9, 22), which was utilizing two 3830 pacing leads with one lead located in the His bundle region as a landmark and another lead seeking the optimal LBBAP pacing site (17). The “nine-partition method” by using single lead has also significantly decreased the mean fluoroscopy time from 12.9 ± 12.9 min to 6.3 ± 3.0 min (15, 16). Like most doctors in China, our group routinely used the single lead technique, which is similar to the nine-partition method, to identify the screwing site of LBBAP lead by using the tricuspid valve annulus as an anatomic marker. Early in the initial stage, we also performed His potential mapping to help to locate the screwing site of the LBBAP lead. Therefore, the prolonged procedure and fluoroscopy time at the initial stage were attributed to His locating, repeated pacing test and fluoroscopy verification of the lead position, and three to five attempts for successful LBBAP. Later in the initial stage, we discarded His locating and directly placed the 3830 lead to the target area just based on the anatomic marker (tricuspid valve annulus). At the stable stage, the whole procedure could be achieved in the right anterior oblique 30° position with one attempt in 80% of patients, which significantly shortened the procedure and fluoroscopy time. Our results could be only interpreted in bradycardia patients with relatively normal cardiac structure because patients indicated for CRT or with congenital heart disease were excluded from our study. The LBBAP procedure might be technically challenging in patients with significantly enlarged right atrium or left ventricle.

Left bundle branch area pacing results in rapid electrical propagation along the conduction system fibers and presents the shortest and constant Sti-LVAT at high and low outputs (14). In our study, LBB potential was less recorded during the stable stage while the mean Sti-LVAT decreased over time. The shortened Sti-LVAT was due to more selective-LBBAP cases achieved with accumulated experience in performing LBBAP. During the stable stage, LBBAP was mainly performed in patients with AVB while patients with SND commonly underwent RVP. LBB potential was less commonly recorded in patients with AVB than patients with SND (76.3 vs. 92.5%) according to Huang et al. (9) and could not be recorded in patients with LBBB without His corrective pacing (23). Moreover, 10.9% of patients during the stable stage underwent the LBBAP procedure without IEGM recording because the device implant in our center was not always performed in a catheter room equipped with a multichannel electrophysiology recording system. The nine-partition method was mainly introduced to perform LBBAP without an electrophysiology recording system (16). LBB potential may not be essential for LBB capture. The previous study has reported that the pQRSd, Sti-LVAT, and LBB capture threshold demonstrate no significant difference between patients with or without LBB potential (24).

The learning curve for LBBAP in our study was fitted based on performance data of one operator because this novel pacing modality has not been widely extended to many doctors in our hospital. The operator performing the LBBAP procedure in our study had implanted more than 600 active-fixation ventricular leads and nearly 60 successful HBP leads. The procedure and fluoroscopy time could be greatly influenced by the experience of the operator. We speculated that operators with high volume experience of HBP might be more skilled in performing LBBAP with shorter procedure and fluoroscopy time when compared with beginners without experience of HBP. The learning curve might be steeper for beginners, but smoother for an experienced operator in HBP. The significant decrease in LBBAP attempts in our study also supported the close association between learning curves and accumulated experience of the operator. Our results may provide novel insights into the routine application of LBBAP for bradycardia patients requiring ventricular pacing.

### Comparison Between RVP and LBBAP

Despite the potential risk of developing cardiac dysfunction, traditional RVP is still the most widely used pacing technique (13). In our study, the procedure and fluoroscopy time between LBBAP at the stable stage and RVP differed statistically significant. However, the absolute difference in the procedure (2.3 min) and fluoroscopy time (1.2 min) might not be clinically significant for ventricular lead implantation. Frequent pacing tests and limited implantation tools might account for the slightly longer time. LBBAP could achieve narrow pQRSd, left ventricular synchrony, and similar pacing parameters to RVP (8, 11, 12). Consistent with previous studies (8, 9, 20), few procedural complications during LBBAP were comparable to RVP. Considering the advantages and disadvantages of the two pacing modalities, LBBAP might be preferred in patients with a high burden of ventricular pacing. However, multicenter large-scale randomized controlled trials are needed to provide evidence for the priority of LBBAP compared with RVP.

## Limitations

Several limitations should be noted. Firstly, different techniques may present various learning curves. Our study described the learning curve of an operator by using the single lead technique for LBBAP. The implant technique was slightly changed in our study with performing His potential mapping in the first less than 20 patients early in the initial stage. However, it should be a neglectable bias because all the rest of the patients underwent the same procedure without His mapping. The improved procedure and fluoroscopy time, illustrated by the fitted learning curve, were associated with accumulated procedure experience instead of changing implant techniques over time. Besides, the learning curve for LBBAP in our study was fitted based on the data of one operator. Because the main point of our study was to explore whether the procedure and fluoroscopy time of LBBAP could be compared with that of RVP or not, the data from an experienced operator in LBBAP should be more convincing than data from several beginners. Furthermore, the number of LBBAP attempts, a sensitive indicator of the experience of operators, decreased significantly over time in our study. The procedure time decreased significantly accompanied by fewer attempts. Finally, safety is also a critical concern for a new technique. The complication events in the LBBAP group were low and only two lead-related complications occurred at the initial stage in the present study. A large sample and multicenter study might be needed to investigate the change of the complication rates in different stages of LBBAP.

## Conclusion

Procedure and fluoroscopy time of LBBAP could be reduced rapidly after 50 cases and plateaued over 150 procedures, while the Sti-LVAT could be shortened further until reaching a plateau after approximately 150 procedures. Compared with RVP, LBBAP can produce a narrower pQRSd and comparable pacing parameters with acceptable procedure and fluoroscopy time.

## Data Availability Statement

The original contributions presented in the study are included in the article/supplementary material, further inquiries can be directed to the corresponding author.

## Ethics Statement

The studies involving human participants were reviewed and approved by Fuwai Hospital, National Center for Cardiovascular Diseases. The patients/participants provided their written informed consent to participate in this study.

## Author Contributions

ZW and HZ contributed to design of the study and performed the statistical analysis. XL organized the database. HZ wrote the first draft of the manuscript. XF wrote sections of the manuscript. All authors contributed to manuscript revision, read, and approved the submitted version.

## Funding

This work was supported by grants (grant no. 81970284) from China's National Natural Science Foundation to XF.

## Conflict of Interest

The authors declare that the research was conducted in the absence of any commercial or financial relationships that could be construed as a potential conflict of interest.

## Publisher's Note

All claims expressed in this article are solely those of the authors and do not necessarily represent those of their affiliated organizations, or those of the publisher, the editors and the reviewers. Any product that may be evaluated in this article, or claim that may be made by its manufacturer, is not guaranteed or endorsed by the publisher.
